# TGF-β Blockade Reduces Mortality and Metabolic Changes in a Validated Murine Model of Pancreatic Cancer Cachexia

**DOI:** 10.1371/journal.pone.0132786

**Published:** 2015-07-14

**Authors:** Stephanie H. Greco, Lena Tomkötter, Anne-Kristin Vahle, Rae Rokosh, Antonina Avanzi, Syed Kashif Mahmood, Michael Deutsch, Sara Alothman, Dalia Alqunaibit, Atsuo Ochi, Constantinos Zambirinis, Tasnima Mohaimin, Mauricio Rendon, Elliot Levie, Mridul Pansari, Alejandro Torres-Hernandez, Donnele Daley, Rocky Barilla, H. Leon Pachter, Daniel Tippens, Hassan Malik, Allal Boutajangout, Thomas Wisniewski, George Miller

**Affiliations:** 1 Department of Surgery, New York University School of Medicine, New York, New York, United States of America; 2 Department of Cell Biology, New York University School of Medicine, New York, New York, United States of America; 3 Department of Neurology, New York University School of Medicine, New York, New York, United States of America; University of South Alabama Mitchell Cancer Institute, UNITED STATES

## Abstract

Cancer cachexia is a debilitating condition characterized by a combination of anorexia, muscle wasting, weight loss, and malnutrition. This condition affects an overwhelming majority of patients with pancreatic cancer and is a primary cause of cancer-related death. However, few, if any, effective therapies exist for both treatment and prevention of this syndrome. In order to develop novel therapeutic strategies for pancreatic cancer cachexia, appropriate animal models are necessary. In this study, we developed and validated a syngeneic, metastatic, murine model of pancreatic cancer cachexia. Using our model, we investigated the ability of transforming growth factor beta (TGF-β) blockade to mitigate the metabolic changes associated with cachexia. We found that TGF-β inhibition using the anti-TGF-β antibody 1D11.16.8 significantly improved overall mortality, weight loss, fat mass, lean body mass, bone mineral density, and skeletal muscle proteolysis in mice harboring advanced pancreatic cancer. Other immunotherapeutic strategies we employed were not effective. Collectively, we validated a simplified but useful model of pancreatic cancer cachexia to investigate immunologic treatment strategies. In addition, we showed that TGF-β inhibition can decrease the metabolic changes associated with cancer cachexia and improve overall survival.

## Introduction

Pancreatic ductal adenocarcinoma (PDA) is an aggressive gastrointestinal cancer, with a five-year survival rate of less than 5%[1]. The majority of patients with PDA present with advanced metastatic disease and have a median survival rate of only 3–6 months[2, 3]. Mortality and poor quality of life in these patients is related to the significant metabolic and nutritional derangements associated with the cancer cachexia syndrome. This syndrome is present in up to 80% of PDA patients and accounts for up to 22% of all cancer-related deaths[4–6].

A prominent feature of cachexia is the development of unintentional weight loss of at least 5% of body mass[7]. The simultaneous loss of both lean body mass and fat mass distinguishes cachexia from starvation, in which lean muscle mass is initially preserved. Cancer cachexia encompasses a host of metabolic derangements including increased energy metabolism–including changes in protein, fat, and glucose metabolism–and immunosuppression with increased release of pro-inflammatory cytokines and acute phase proteins[6]. In addition, neurocognitive effects of cachexia include fatigue, impaired memory and cognition, and decreased physical activity secondary to increased resting energy expenditure[8, 9]. Collectively, these derangements reduce the quality of life in cachectic patients and may even contribute to a decreased therapeutic response to chemotherapy[10].

Unfortunately, there are few evidence-based effective treatment strategies for cancer cachexia. Because this disease process encompasses a variety of host imbalances, effective treatments should ideally target multiple metabolic pathways. Cachexia has been treated with use of progestins such as megestrol acetate and medroxyprogesterone acetate to increase appetite and weight gain along with corticosteroids to improve mood and reduce inflammation[11, 12]. However, the clinical benefits of these treatments have been marginal[11, 13]. Other potential therapeutic agents that have been studied include cyclooxygenase (COX-2) inhibitors and investigational drugs including ghrelin and ghrelin mimetics, combined tumor necrosis factor alpha (TNF-α) and interleukin 6 (IL-6) inhibitors, as well as β-adrenoceptor agonists and myostatin inhibitors[6]. However, effective clinical results in cancer cachexia patients have been elusive.

Validated animal models of pancreatic cancer cachexia are necessary to develop effective immunotherapy-based treatment strategies. Cachexia-inducing cell lines such as Lewis Lung Carcinoma (LLC) and colorectal tumors have been used widely in animal models of cancer cachexia[4, 14]. These tumor lines are implanted subcutaneously in experimental animals and are allowed to grow until symptoms of cachexia are achieved. Such heterotopic models have also been used in studying cachexia in PDA[15, 16]. However, a primary limitation of these models is their inability to metastasize[17]. Xenograft models of human PDA in which human PDA cell lines are implanted into athymic or immunosuppressed mice have also been employed[17]. However, although xenograft models effectively mimic tumor/host epigenetics and can metastasize, they markedly alter the immunologic milieu and therefore hinder the ability to study the immunologic changes associated with cachexia as well and any immunotherapeutic strategies[17, 18].

Potential immunologic targets for cachexia treatment in PDA, include Toll-like receptors (TLRs) and transforming growth factor beta (TGF-β). Toll-like receptors are a family of pattern recognition receptors on innate immune cells which directly link environmental stimuli to innate inflammatory responses[19]. TLRs can be activated by ligating non-pathogenic ‘danger molecules’, known as DAMPs, including by-products of inflammatory injury and cellular necrosis[20, 21]. TLRs have been implicated to play a significant role in PDA, as our lab has recently shown that TLR7 ligation accelerates pancreatic carcinogenesis and that TLR7 blockade protected against tumor progression[22]. Additionally, TLR9 stimulation has been correlated with the invasive and metastatic potential of human PDA, thereby implicating this receptor as potential target in the treatment of this disease[23]. However, to our knowledge neither TLR7 nor TLR9 has been studied in the treatment of pancreatic cancer cachexia.

The transforming growth factor beta (TGF-β) pathway plays an important role in cell differentiation and inflammation[24]. TGF-β receptor activation leads to phosphorylation of Smad proteins including Smad2, Smad3, and Smad4, which in turn regulate the transcription of cell cycle inhibitors, including p21[25]. In PDA, TGF-β has paradoxically been shown to act as both a tumor suppressor and tumor promoter[26]. TGF-β can inhibit proliferation, suppress transformation, and decrease PDA progression[27]. However, at the same time TGF-β blockade may reduce the invasiveness of PDA[28]. Nonetheless, mutations in downstream mediators of TGF-β signaling, including Smad4 have been shown to promote PDA progression[29]. Further, although TGF-β has been implicated to promote cancer cachexia[30, 31], to our knowledge, TGF-β blockade has not been studied as a therapeutic strategy in the treatment of pancreatic cancer cachexia. The purpose of this study was to develop a syngeneic metastatic model of pancreatic cancer cachexia and to investigate immunotherapeutic treatment strategies, including TLR 7/9 blockade and TGF-β blockade.

## Materials and Methods

### Animals, cell lines, and in vivo experiments

Male C57BL/6(H-2k^b^) and Toll-like receptor 9 knockout (TLR9^-/-^) mice were purchased from the Jackson Laboratory (Bar Harbor, ME). Pdx1^Cre^;Kras^LSL-G12D^;Tp53^R172H^ (KPC) mice were a gift of Mark Philips (New York University)[32]. Animals were bred in house and age-matched 6–8 week old mice were used for all experiments. The Pan02 murine PDA cell line (gift of Daniel Meruelo, New York University), which is syngeneic to C57BL/6, was grown in complete RPMI culture medium supplemented with 10% fetal bovine serum and 1% penicillin-streptomycin. FC1242 PDA tumor cells derived from KPC mice were a gift from David Tuveson (Cold Spring Harbor Laboratory). Mice were injected i.p. with either 10 million Pan02 or 5 million FC1242 cells to induce cancer cachexia syndrome. Selected cohorts were additionally administered 2.6 mg/kg of the Toll-like receptor 7 and 9 (TLR7/9) inhibitor IRS 954 (Dynavax, Berkeley, CA), thrice weekly, or 200 μg of transforming growth factor beta (TGF-β) inhibitor (clone 1D11.16.8, inhibits TGF- β_1_, β_2_, and β_3_) (Bioxcell, West Lebanon, NH) twice weekly, beginning immediately after inoculation with Pan02 and continuing for the duration of the experiment. Animals were monitored twice daily. Mice were sacrificed using CO2 narcosis followed by cervical dislocation at the end of the 60-day study period, or earlier if they appeared moribund or exhibited greater than 20% weight loss. To induce subcutaneous tumor growth, mice were injected in the right flank with 10 million Pan02 cells. Tumor growth was measured using a caliper twice weekly. Levels of cytokines in serum were determined using a cytometric bead array (BD Biosciences). The New York University School of Medicine IACUC approved all procedures.

### Measurement of weight and body composition

Weight and body composition including: fat mass, lean mass, % body fat, bone mineral content, and bone mineral density were measured weekly using dual-energy x-ray absorptiometry (DEXA) with the Lunar PIXImus (PIXImus, Fitchburg, WI) device for small animals. All mice were anesthetized prior to DEXA data collection using 0.1 mg/g Ketamine and 0.01 mg/g Xylazine by i.p. injection. Data was analyzed using the Lunar PIXImus software version 1.45. Arm circumference was calculated as 3.14 times the upper arm diameter, as measured using a caliper with accuracy ± 0.1mm. Subscapular skinfold thickness was also measured using a caliper.

### Western blotting and immunohistochemistry

For Western blotting, total protein was isolated from mouse quadriceps or adipose tissue by homogenization in RIPA buffer with complete protease inhibitor cocktail and phosphatase inhibitor cocktail (Roche, Pleasanton, CA)[33]. Protein quantification was determined by the Bradford protein assay, and samples were equally loaded on 10% polyacrylamide gels (NuPage, Grand Island, NY), electrophoresed at 200V, electrotransferred to PVDF membranes, and probed with antibodies to Atrogin-1 (1:500; ECM Biosciences, Versailles, KY), Myostatin (1:200; Abcam, Cambridge, MA), MuRF1, ZAG (1:500; both Santa Cruz Biotechnology, Dallas, TX), p21 (1:200; Santa Cruz), β-actin, Smad2, p-Smad2/3 (1:1000; all Cell Signaling, Danvers, MA), TGF-β (1:1000; Abcam), and Ezrin (1:1000; BD Transduction Laboratories, San Jose, CA). Quantification was performed by measuring integrated density and normalizing to loading controls. For immunohistochemical analysis, paraffin-embedded tissue sections were stained using polyclonal anti-TGF-β (1:100; Abcam, Cambridge, MA), anti-p21 (1:100, Santa Cruz), or anti p-Smad 2/3 (1:50, Santa Cruz) and the corresponding isotype controls[33].

### qPCR and cellular proliferation Assay

For PCR analysis, total RNA was isolated using the RNEasy Mini Kit (Qiagen, Germantown, MD) and cDNA was made using the High Capacity Reverse Transcription Kit (Applied Biosystems, Grand Island, NY). RT-PCR was performed on a Stratagene Mx3005P QPCR System (Agilent Technologies) using pre-designed primers for mouse MuRF1 and Atrogin-1 (both Qiagen, Germantown, MD)[34]. In vitro tumor cell proliferation was assessed using the XTT II assay according to the manufacturer’s protocol (Cell Proliferation Kit II, Roche, Pleasanton, CA) and expressed as % proliferation compared to control (100%).

### Neurocognitive Assays

For the rotarod test, animals were placed onto a rod (diameter 3.6 cm) apparatus to assess differences in motor coordination and balance by measuring forelimb and hindlimb motor coordination and balance (Rotarod 7650 accelerating model; Ugo Basile, Varese, Italy) as we have previously described[35]. Each animal was tested for three sessions, with each session separated by 15 min, and measures were taken for rotarod speed when the animals fell from the top of the rotating barrel.

The spontaneous object recognition test (ORT) was conducted in a square-shaped open field box, raised 50 cm from the floor as we have previously described[35]. Two novel objects were placed at diagonal corners in the open field and the animal was allowed to explore for 15 min. For any given trial, the objects in a pair were 10 cm high and composed of the same material so that they could not readily be distinguished by olfactory cues. The time spent exploring each object was recorded by a tracking system (San Diego Instruments, San Diego, CA), and at the end of the training phase the mouse was removed from the box for the duration of the retention delay (3 h). During retention tests, the animals were placed back into the same box, in which one of the previous familiar objects used during training was replaced by a novel object, and were allowed to explore freely for 6 min. A different object pair was used for each trial for a given animal, and the order of exposure to object pairs as well as the designated sample and novel objects for each pair were counterbalanced within and across groups. The time spent exploring the novel and familiar objects was recorded for 6 min. The object recognition test index was calculated as the ratio of time spent exploring the novel object to the time spent exploring both the novel and familiar object.

### Statistics

Data is presented as mean ± standard error. Survival was measured according the Kaplan-Meier method. Statistical significance was determined by the Student’s *t* test and the log-rank test using GraphPad Prism 6 (GraphPad Software, La Jolla, CA). P-values <0.05 were considered significant.

## Results

### Development of a syngeneic model of advanced pancreatic cancer

**Fig 1:**
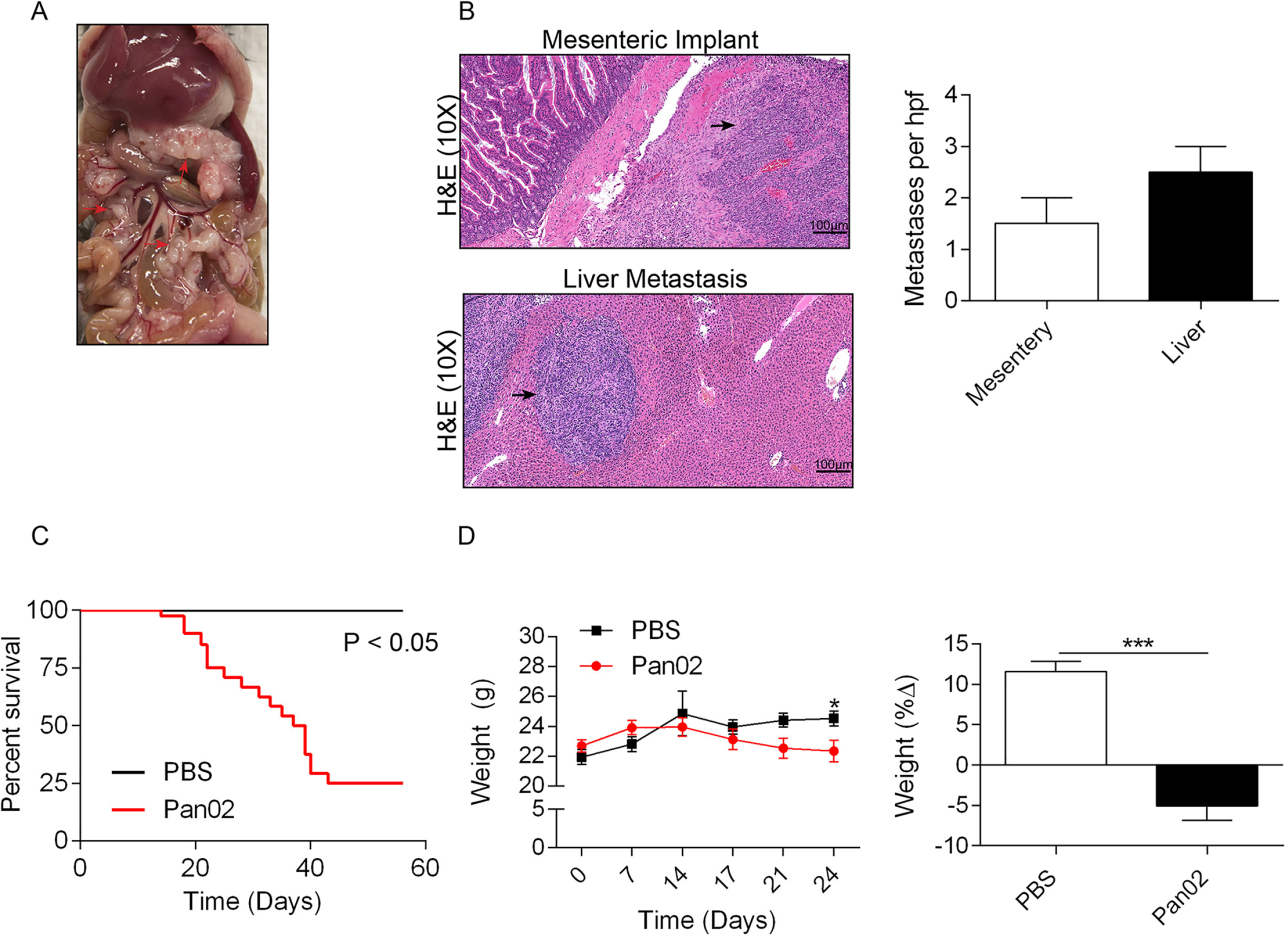
Mice challenged with Pan02 developed lethal tumor burden associated with progressive weight loss. **(A)** Mice administered Pan02 exhibited gross and **(B)** microscopic evidence of peritoneal carcinomatosis as well as microscopic liver implants. Representative images and summary data are shown. **(C)** Kaplan-Meier analysis of survival was performed in PBS and Pan02 treated animals (n = 20/group, p<0.05). **(D)** The average body weights of mouse cohorts treated with PBS or Pan02 were calculated at various time points as was the total weight change over the course of the study (n = 20; *p<0.05, ***p<0.001).

Mice administered Pan02 PDA cells by i.p injection developed overt peritoneal carcinomatosis (Fig 1A) with implants on peritoneal surfaces including the small bowel mesentery as well as microscopic liver metastases (Fig 1B) within three weeks after treatment. These findings correlate with advanced PDA in humans, which is associated with liver and/or peritoneal metastases in nearly all cases[36]. Approximately 75% of Pan02-treated animals died within 45 days after treatment (Fig 1C). Further, consistent with PDA cachexia syndrome, Pan02-treated mice exhibited progressive weight loss beginning approximately 2 weeks after tumor challenge (Fig 1D).

### Evidence of altered body composition

**Fig 2:**
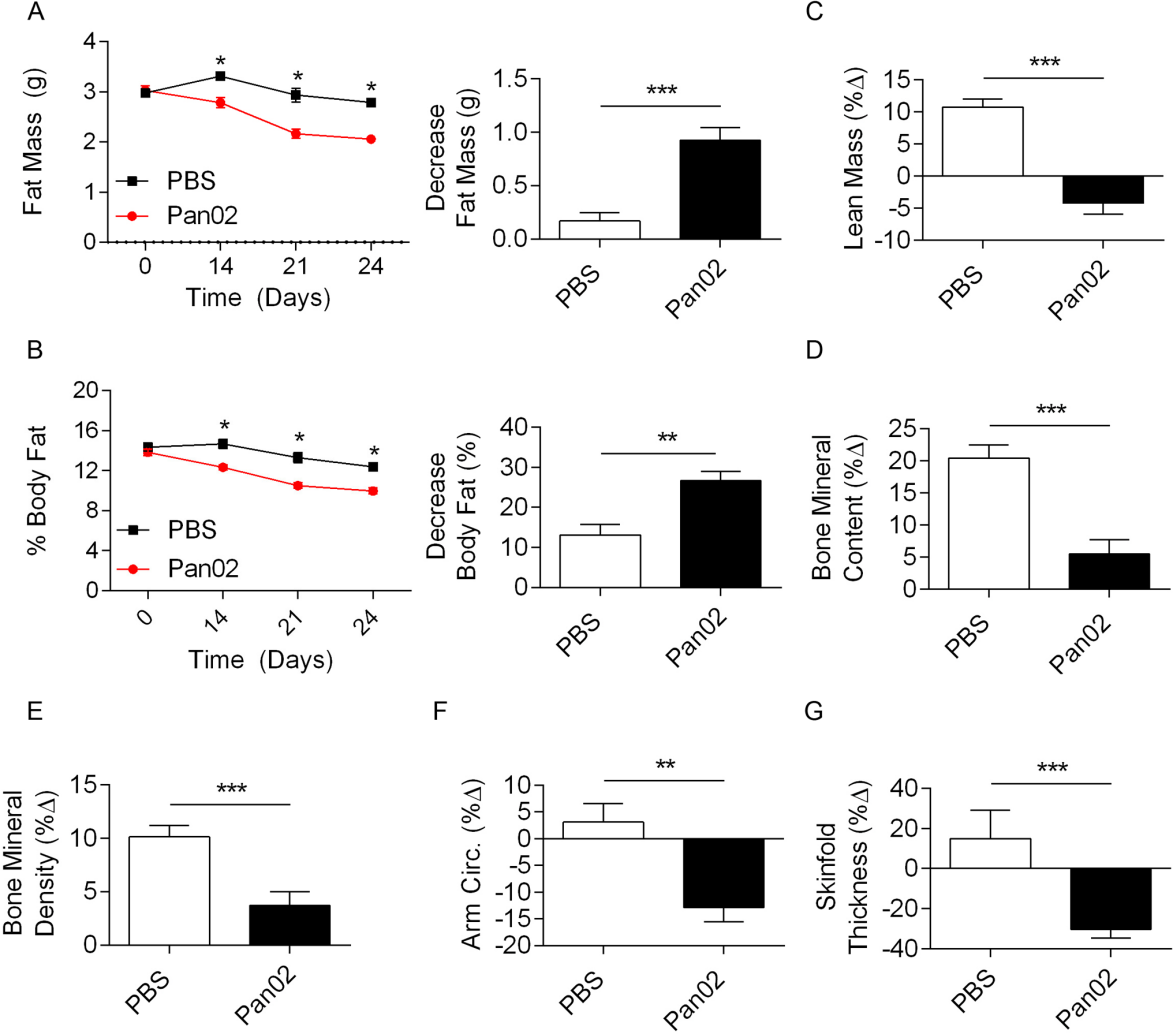
Mice challenged with Pan02 developed evidence of cachexia based on body composition. **(A)** Average fat mass **B)** and average percent body fat of mouse cohorts treated with PBS or Pan02 was calculated at various time points as well as total change over the course of the study. **(C)** Changes in lean mass, **(D)** bone mineral content, **(E)** bone mineral density, **(F)** arm circumference, and **(G)** skinfold thickness were also calculated (n = 20; *p<0.05, **p<0.01, ***p<0.001).

Clinical pancreatic cancer cachexia is associated not only with weight loss, but also with loss of fat, muscle, and bone content[6, 37]. Accordingly, we found that Pan02-treated mice lost nearly 1g in fat mass by 24 days (Fig 2A), and had significantly reduced percent body fat as compared to control mice (Fig 2B). Tumor-bearing mice also exhibited lower lean body mass (Fig 2C), and reduced elevations in both bone mineral content (Fig 2D) and bone mineral density as compared with control mice (Fig 2E). To further quantify body compositional changes, we measured upper arm circumference (Fig 2F) and subscapular skinfold thickness (Fig 2G), and found both to be markedly diminished in the Pan02-treated mice. Taken together, these findings correlate with the metabolic changes associated with clinical pancreatic cancer cachexia syndrome.

### Evidence of muscle atrophy and inflammatory change

**Fig 3:**
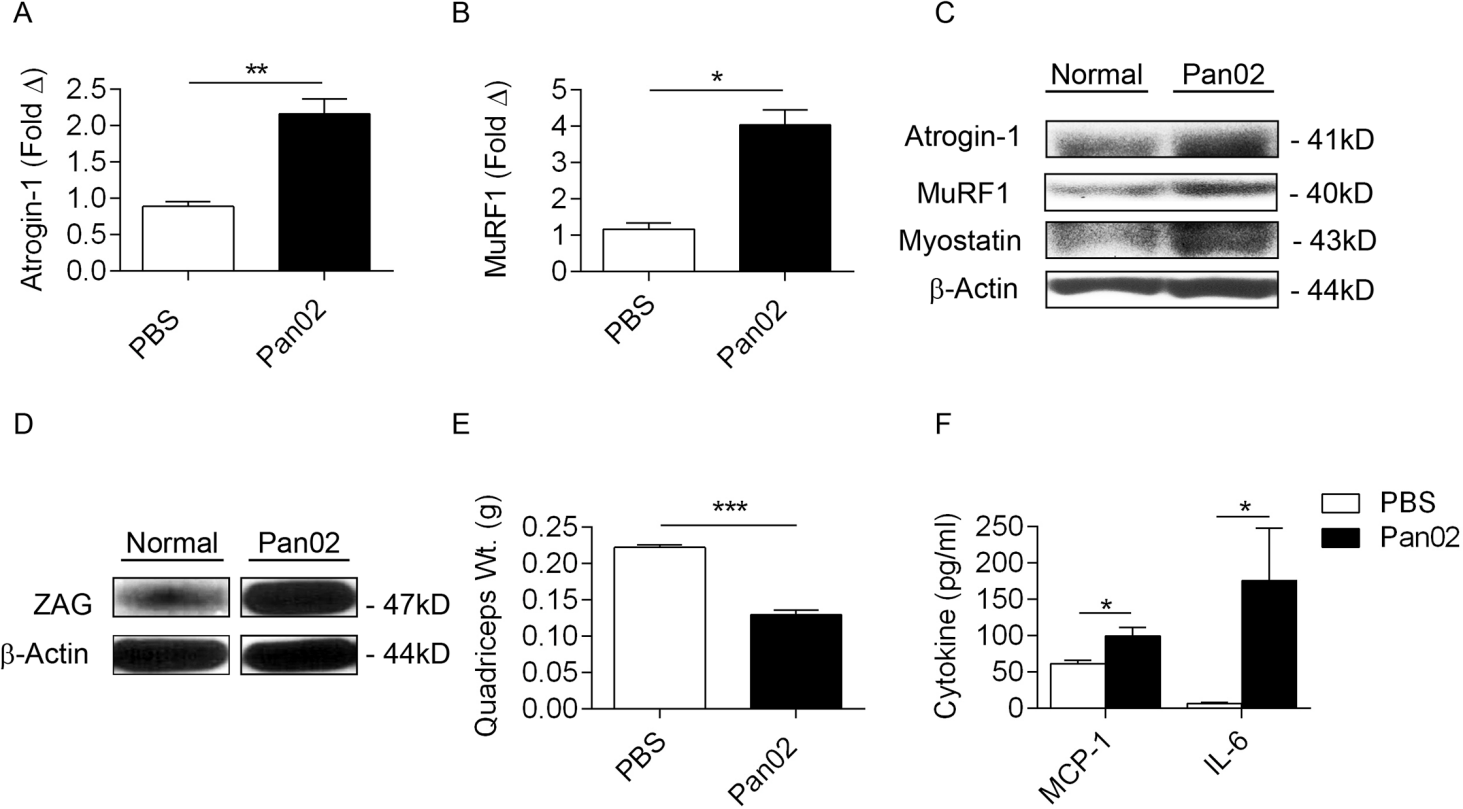
Evidence of muscle wasting and systemic inflammation. **(A)** mRNA levels of Atrogin-1 and **(B)** MuRF1 in quadriceps muscles of mice treated with PBS or Pan02 were calculated by qPCR. **(C)** Similarly, levels of Atrogin-1, Myostatin, MuRF1, and β-actin in quadriceps muscle were calculated by Western blotting. **(D)** ZAG expression was tested in visceral adipose tissue from cohorts of mice treated with either PBS or Pan02. **(E)** Weight of quadriceps muscles from mouse cohorts treated with PBS or Pan02 was calculated. **(F)** Serum levels of MCP-1 and IL-6 were compared in PBS- and Pan02-treated mice (n = 5; *p<0.05, **p<0.01, ***p<0.001).

Since proteolysis and muscle atrophy are hallmarks of human pancreatic cancer cachexia[38], we measured markers of muscle atrophy in both Pan02-treated mice and control mice. Both atrogin-1 and MuRF-1 are E3 ubiquitin ligases expressed in skeletal muscle which target proteins for proteolysis[39, 40].We found increased intramuscular levels of atrogin-1 (Fig 3A) and MuRF-1 (Fig 3B) in the tumor-bearing mice by qPCR. Western blotting confirmed these results and additionally showed elevated muscular expression of myostatin in treated animals (Fig 3C), suggestive of intramuscular proteolysis[41]. Similarly, zinc alpha glycoprotein (ZAG), a marker of lipolysis, was also markedly elevated in adipose tissue of Pan02-treated mice (Fig 3D). Consistent with a proteolytic state, quadriceps weight was lower in tumor-bearing mice (Fig 3E). Patients with advanced pancreatic cancer-cachexia commonly exhibit elevated pro-inflammatory cytokines, which are associated with poor survival[42, 43]. Accordingly, levels of MCP-1 and IL-6 were elevated in the serum of tumor-bearing hosts (Fig 3F).

### Combined TLR7/9 inhibition does not mitigate pancreatic cancer cachexia

**Fig 4:**
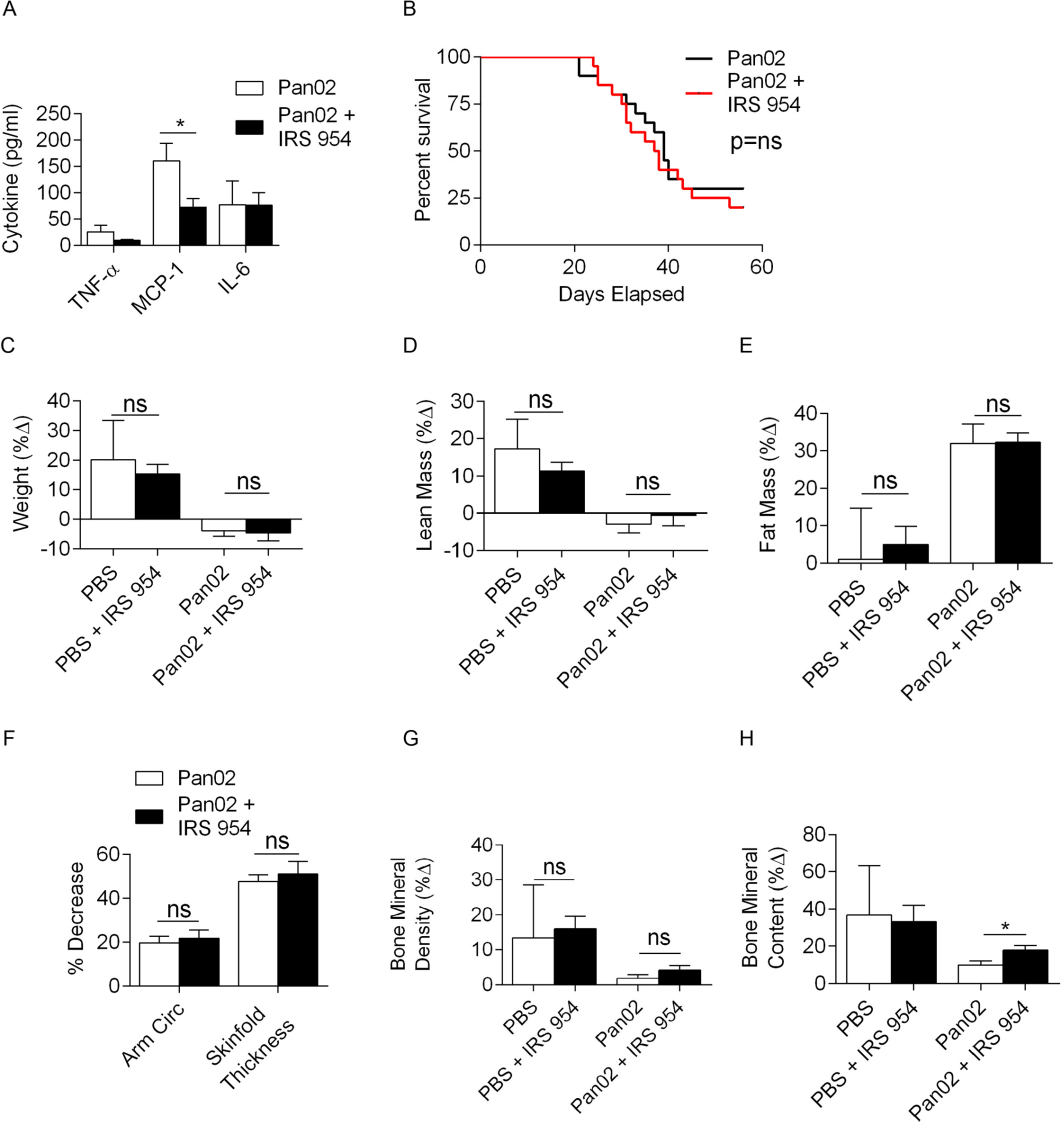
Combined TLR7/9 blockade mitigates inflammation but does not substantially improve the cachectic phenotype. **(A)** Mice were treated with Pan02 or Pan02 + IRS 954 and tested for serum cytokines (n = 20; *p<0.05) and **(B)** survival using the Kaplan-Meier method (n = 20; p = ns). **(C)** Similarly, mice were treated with PBS, IRS 954, Pan02, or Pan02 + IRS 954 and tested for overall weight change, and changes in **(D)** lean mass, **(E)** fat mass, **(F)** arm circumference and skinfold thickness, **(G)** bone mineral density, **(H)** and bone mineral content (n = 20/group; *p<0.05).

We previously reported that Toll-like receptor (TLR) ligation accelerates pancreatic cancer progression whereas blockade of select TLR signaling pathways can be protective[22, 44]. Therefore, we postulated that TLR inhibition may be protective against pancreatic cancer cachexia. To test this, mice were treated with IRS 954, a combination TLR7/9 oligonucliotide inhibitor[45]. As anticipated, selected serum cytokines were lower in IRS 954 treated mice (Fig 4A). However, animals treated with Pan02 + IRS 954 had a similar overall survival compared to mice treated with Pan02 alone (Fig 4B). Moreover, there were no significant differences in cumulative weight loss (Fig 4C), changes in lean body mass (Fig 4D), fat mass (Fig 4E), arm circumference and skinfold thickness (Fig 4F), or bone mineral density (Fig 4G) between the treated and control groups. However, IRS 954-treated tumor-bearing mice did have higher bone mineral content than untreated tumor-bearing mice (Fig 4H). Genetic deletion of TLR9 was similarly ineffective at alleviating pancreatic cancer cachexia in Pan02-challanged animals (S1 Fig). Taken together, our experiments employing selective or combined TLR inhibition did not substantively improve experimental pancreatic cancer cachexia.

### Treatment with TGF-β inhibitor protects against pancreatic cancer cachexia

**Fig 5:**
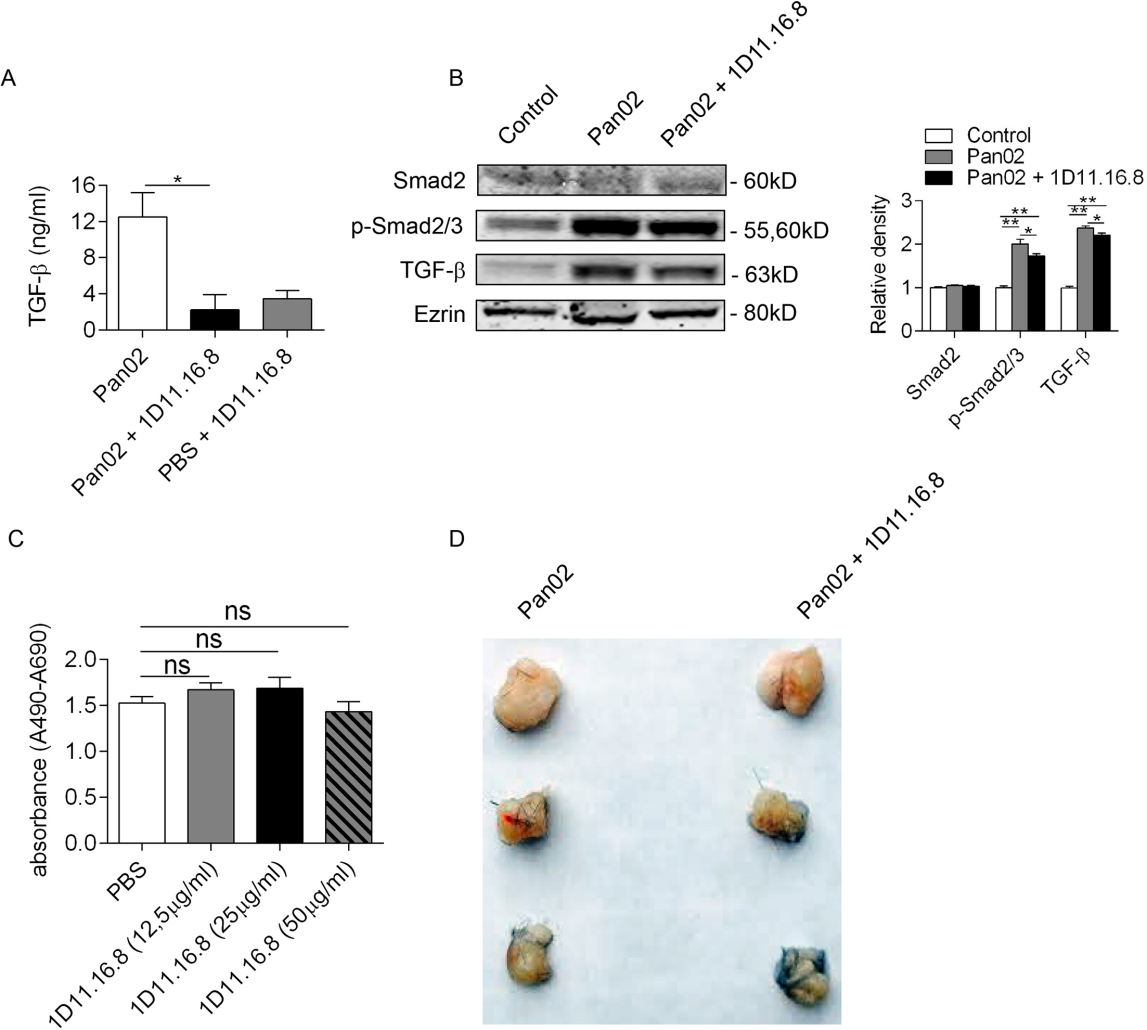
Effects of TGF-β inhibition on pancreatic cancer growth. **(A)** Serum levels of TGF-β were measured in mice treated with Pan02, Pan02 + 1D11.16.8, or PBS + ID11.16.8 (n = 10/group; *p<0.05). **(B)** Intramuscular levels of Smad2, p-Smad2/3, TGF-β, and Ezrin (a loading control) were calculated by Western blotting. Density analysis was done based on triplicates (*p<0.05, **p<0.01, ***p<0.001). **(C)** Pan02 cell proliferation was measured after treatment with increasing doses of 1D11.16.8. **(D)** 10^7^ Pan02 cells were implanted subcutaneously into the right flank of mice treated with either PBS or 1D11.16.8. Tumors were measured twice weekly by caliper and explanted tumors were compared for tumor size (n = 5/group).

The TGF-β superfamily has an important role in tumor immune responses[46]. TGF- β has also been implicated in cachexia by stimulating muscle breakdown and proteolysis[47]. However, targeting TGF-β in pancreatic cancer cachexia has not been tested. We therefore first determined if TGF-β blockade was relevant in our pancreatic cancer cachexia model. TGF-β and the downstream transcriptions factor p21 and p-Smad 2/3 were highly expressed by Pan02 tumors as well as in endogenous models of advanced murine pancreatic cancer (S2 Fig). We confirmed that mice harboring Pan02 tumors treated with a neutralizing TGF-β mAb (1D11.16.8) exhibited lower serum levels of TGF-β (Fig 5A), as well as lower levels of TGF-β and downstream signaling marker p-SMAD2/3 in skeletal muscle (Fig 5B). Further, treatment of Pan02 tumor cells with 1D11.16.8 did not alter their cellular proliferation in vitro (Fig 5C) or subcutaneous tumor growth in vivo (Fig 5D). Lastly, we found that treatment of mice with 1D11.16.8 alone in non-tumor-bearing hosts did not alter weight change, lean body mass, fat mass, arm circumference, or bone mineral density (S3 Fig).

**Fig 6:**
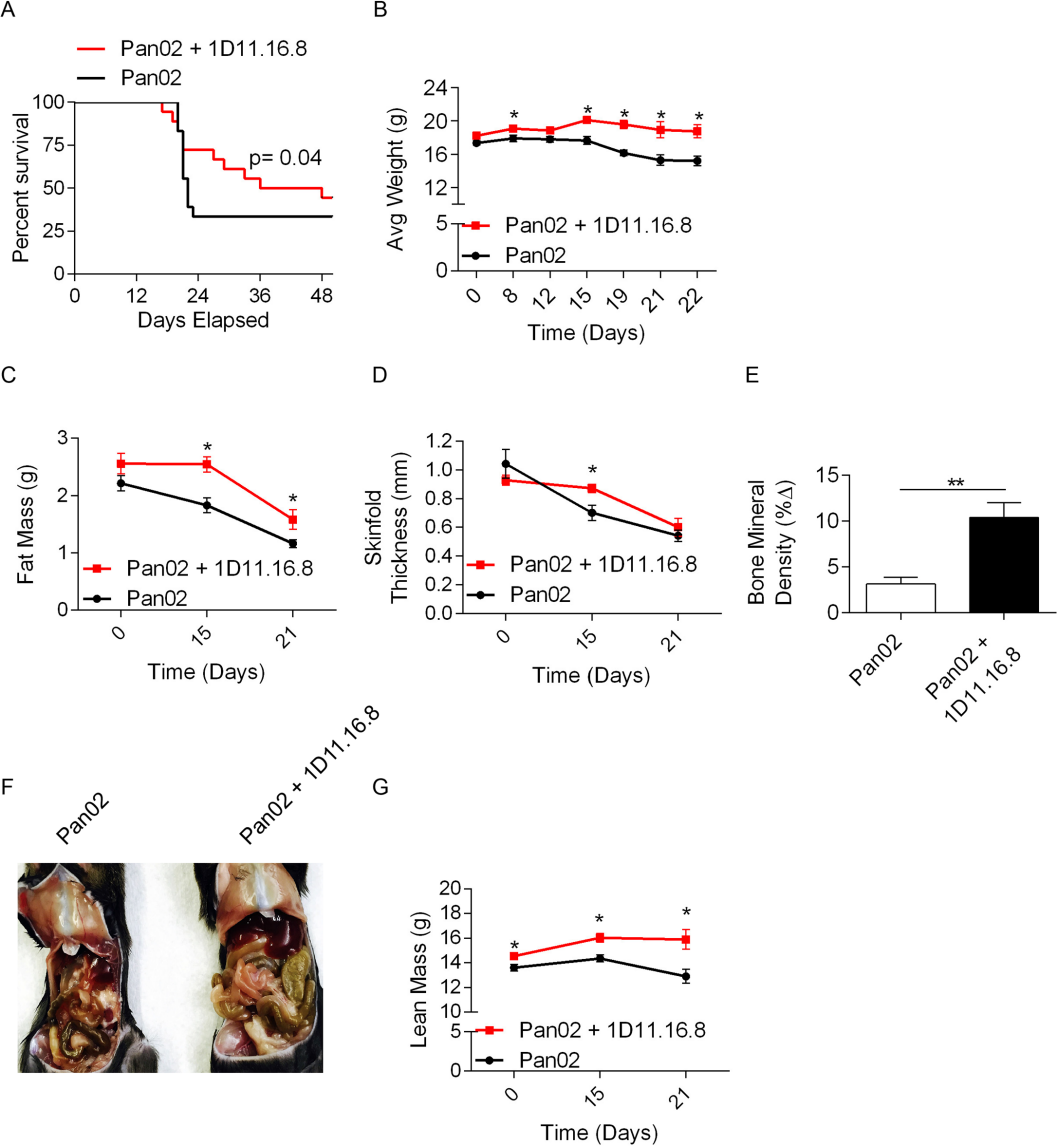
TGF-β inhibition improves overall survival and body composition in pancreatic cancer cachexia. **(A)** Mice were treated with Pan02 or Pan02 + 11D1.16.8 and tested for survival using Kaplan-Meier analysis. **(B)** Cohorts of mice were also tested for weight change, and changes in **(C)** fat mass, **(D)** skinfold thickness, or **(E)** bone mineral density (n = 10/group; *p<0.05, **p<0.01). **(F)** Mice were treated with Pan02 or Pan02 + 11D1.16.8 and were evaluated for gross loss of lean body mass. **(G)** Cohorts of mice were also tested for change in lean body mass (n = 10/group; *p<0.05).

Next, we investigated whether pharmacologic blockade of TGF-β could mitigate pancreatic cancer cachexia. Mice treated with Pan02 + 1D11.16.8 exhibited improved survival (Fig 6A) and significantly less weight loss (Fig 6B) compared to control mice. Tumor-bearing mice treated with 1D11.16.8 also had higher fat mass (Fig 6C), skinfold thickness (Fig 6D), and bone mineral density (Fig 6E) compared with controls. Further, we found significantly diminished evidence of proteolysis in mice treated with 1D11.16.8, which is reflected by a noticeable difference in body contour as compared to control mice (Fig 6F). Accordingly, there was markedly attenuated reduction in lean body mass in mice treated with 1D11.16.8 (Fig 6G). Additionally, we also confirmed that 1D11.16.8 effectively attenuates pancreatic cancer cachexia using KPC-derived FC1242 cells. Specifically, we found that FC1242 mice treated with 1D11.16.8 also had improved overall survival, decreased fat mass loss, higher bone mineral density, and decreased loss of body fat percentage as compared to control mice (S4 Fig).

Cachexia can be associated with motor and cognitive dysfunction [48]. On assessment of motor impairment we noted improved function in tumor bearing mice treated with TGF-β inhibitor as measured by higher rotarod running speed compared with mice administered Pan02 alone. Conversely, the slight improvement in the object recognition test in Pan02-bearing mice that were treated with 1D11.16.8 did not reach statistical significance (S5 Fig). Collectively, these data suggest that targeting TGF-β is an attractive strategy for targeting pancreatic cancer cachexia.

## Discussion

We validated a syngeneic murine model of metastatic PDA cachexia through peritoneal injection of the Pan02 cell line. Mice developed evidence of peritoneal metastases and liver implants, which corresponds with clinical PDA[1]. Additionally, the success rate of this model is close to 100% with nearly all Pan02-treated mice developing cancer cachexia syndrome as evidenced by significant weight loss, decreased fat and lean body mass, proteolysis, and lipolysis.

In the current study, we show that neither genetic deletion of TLR9 nor blockade of TLR7/9 using IRS 954 significantly altered the metabolic changes associated with pancreatic cancer cachexia. To our knowledge this is the first study to specifically investigate the efficacy of TLR blockade for the treatment of cachexia in PDA, although we and others have previously shown that TLRs play an important role in the pathogenesis of pancreatic cancer[49]. For example, Schwartz et al. showed that TLR3 was constitutively expressed in human pancreatic cancer cell lines, and TLR blockade with phenylmethimazole inhibited pancreatic cancer tumor growth in vivo[26]. Additionally, LPS activation of TLR4/MyD88 signaling has been shown to play an important role in pancreatic cancer tumor growth and migration, and blockade of this pathway decreases tumor invasive ability[29]. Further, our lab has previously shown that activation of TLR4 and TLR7 accelerates pancreatic carcinogenesis[22, 50]. Others have shown that TLR9 correlates with the invasive and metastatic potential of human pancreatic cancer cell lines[23].

Based on the aforementioned data and the fact that TLRs are central mediators of pro-inflammatory cytokine production, including TNF-α and IL-6, which play an important role in the pathogenesis of cancer cachexia[43, 51], we hypothesized that TLR blockade may be therapeutic in pancreatic cancer cachexia. The cytokines released after TLR activation may theoretically contribute to cancer cachexia syndrome by inducing proteolysis, lipolysis, and other metabolic changes through NF-κβ transcription[52]. In advanced pancreatic cancer, pro-inflammatory cytokines are produced from the cancer cells themselves, as well as by the liver as part of the acute phase response. Interestingly, increased cytokine levels are thought to be poor prognostic factors in cachexia, as studies have shown that IL-6 polymorphisms and elevated levels of IL-6 are associated with increased cachexia and decreased survival in pancreatic cancer patients[53]. Our model of pancreatic cancer cachexia was associated with marked elevations in serum levels of IL-6. However, IL-6 remained elevated after TLR7/9 blockade. More importantly, neither TLR7/9 blockade nor genetic deletion of TLR9 improved overall mortality or the metabolic changes of pancreatic cachexia in our validated mouse model. These negative findings may suggest that further downstream targeting of Toll-like receptor pathways, such as NF-κβ signaling, are required to alter the effects of cachexia.

Most critically, we found that mice treated with TGF-β inhibitor had improved overall survival and were protected from cancer cachexia as measured by weight loss, lean body mass, fat mass, skinfold thickness, and bone mineral density. We also showed that treatment with 1D11.16.8 decreased proteolysis and induced limited improvement in neurocognitive impairment in our Pan02 mouse model, as evidenced by decreased lean body mass and improved scores in rotarod running speed. Indeed neurocognitive impairments have been well-described in cachectic patients[48, 54, 55]. Furthermore, we showed that the effects of TGF-β blockade on cachexia are not specific to Pan02 tumor hosts, since we validated our results in a KPC-derived PDA animal model.

The TGF-β superfamily, including TGF-β_1_, TGF-β_2_, and TGF-β_3,_ is involved in diverse cellular processes including cell growth and differentiation, angiogenesis, immunosuppression, apoptosis, and cellular homeostasis[25]. Additionally, TGF-β plays an important role in tumor suppression through inhibition of cell growth by inducing the cell cycle inhibitor, p21[56]. The Smad proteins, are the main family of transcription factors which propagate TGF-β signaling. The Smad family consists of R-Smads, Smad 2 and Smad 3 which after phosphorylation, associate with the Co-Smad, Smad 4, and enter the nucleus to activate transcription[26]. Interesting, we showed that both p21 and Smad 2/3 were highly upregulated in both our Pan02 and KPC transgenic mouse models of pancreatic cancer. However, we found that the effect of TGF-β inhibition on cachexia was not related to inhibition of tumor growth, as evidenced by no difference in vitro Pan02 cellular proliferation or subcutaneous tumor growth.

Our study adds to a wealth of research which investigates the role of TGF-β in PDA. Due to its effect on many cellular processes, alterations in TGF-β signaling have been implicated to have an important role in the pathogenesis and progression of many cancers[26, 57, 58]. It is widely accepted, however, that TGF-β has roles as both a tumor suppressor and tumor promoter in PDA depending on the tumor stage and cellular milieu[24, 59]. For example, nearly 100% of pancreatic tumors have a mutation in at least one of the TGF-β pathway genes, and over 50% have gene deletion in SMAD4 (DPC4) [26]. However, at the same time, high serum levels of TGF-β_1_ have been associated with an increased risk of PDA development, and PDA patients with low nuclear staining of TGF-βR2 and high TGF-β_1_ levels may have lower overall survival[58, 60]. Furthermore, SMAD4 appears to play a key role in the progression of PDA tumorogenesis through effects on the tumor microenvironment. However, the precise role of TGF-β in this disease process remains controversial. For example, multiple studies have shown that TGF-β inhibition promotes PDA progression[27, 57], whereas others have shown that in certain tumors SMAD4 facilitates epithelial to mesenchymal transition and tumor growth[29]. Nonetheless, despite these paradoxical data, inhibition of TGF-β signaling in PDA is an important area of interest, which has shown therapeutic promise. For example, Melisi et al. showed that LY2109761, an inhibitor of TGF-β receptors I and II reduced metastasis of PDA in vivo[61]. Additionally, Kim et al. showed that treatment with a TGF-β_1_ inhibitor sensitized drug-resistant PDA cells to gemcitabine[62].

Although there is a wealth of research regarding the role of TGF-β signaling in the pathogenesis of PDA, to our knowledge there are no other studies evaluating the direct role of TGF-β blockade as a treatment for cachexia in this disease. Previous studies have demonstrated that TGF-β_1_ can induce cachexia and anorexia in mice[30, 31]. More specifically, TGF-β is a potent inducer of muscle atrophy through activin, inhibin, and myostatin, all part of the TGF-β protein family, which are shown to have a significant role in cachexia[63]. For example, inhibin-deficient mice develop adrenal tumors and cachexia[64], and myostatin administration induces cachexia[41]. Additionally, Zhou et al. recently showed that pharmacological blockade of ActRIIB, an activin, can reverse cancer cachexia and muscle wasting in a mouse model with C26 colon cancer[47]. Furthermore, atrogin and MuRF1 are E3 ubiquitin ligases associated with proteolysis and sarcopenia which are induced in response to myostatin/ TGF-β signaling through Smad2/3[65–68]. In the current study we showed that atrogin-1, myostatin, and MuRF1 were upregulated in our model, indicating that TGF-β inhibition likely altered proteolysis through these mediators of skeletal muscle hypertrophy, and possibly through Smad2/3.

In summary, we have developed a validated, simple, syngeneic mouse model of pancreatic cancer cachexia which can be an important platform to test novel agents, including immunotherapies. Further, we have implicated a role for TGF blockade in the treatment of this disease. We have shown improved overall survival and reduced metabolic changes associated with pancreatic cancer cachexia in animals treated with TGF-β inhibitor and suggest a promising role for TGF-β blockade as a therapeutic strategy in this otherwise terminal process.

## Supporting Information

S1 Fig TLR9 deletion does not protect against pancreatic cancer cachexia. **(A)** WT and were treated TLR9^-/-^ mice were treated with PBS or Pan02 and tested for overall weight change, and changes in **(B)** lean body mass, **(C)** fat mass, **(D)** bone mineral density, **(E** bone mineral content, and **(F)** arm circumference (n = 10/group).
(TIF)

S2 Fig TGF-β expression in pancreatic carcinoma tissues **(A)** Expression of TGF-β was tested by immunohistochemistry in Pan02 peritoneal carcinomatosis and **(B)** in the pancreata of KPC mice. **(C)** Expression of p21 was tested by immunohistochemistry in Pan02 peritoneal carcinomatosis and **(D)** in the pancreata of KPC mice. **(E)** Expression of p-Smad2/3 was tested by immunohistochemistry in Pan02 peritoneal carcinomatosis and **(F)** in the pancreata of KPC mice. **(G)** Protein levels of p21 in quadriceps muscle of mice treated with PBS and Pan02 were tested by Western blotting (n = 5/group).
(TIF)

S3 Fig Effects of TGF-β inhibition on control mice. **(A)** Mice were treated with PBS or 11D1.16.8 and tested for weight change, and changes in **(B)** lean mass, **(C)** fat mass, **(D**) arm circumference, or **(E)** bone mineral density (n = 10/group).
(TIF)

S4 Fig TGF-β inhibition improves overall survival and body composition in a second model of pancreatic cancer cachexia. **(A)** Mice were treated with FC1242 or FC1242 + 11D1.16.8 and tested for survival using Kaplan-Meier analysis. **(B)** Cohorts of mice were also tested for change in fat mass, **(C)** change in bone mineral density, and **(D)** body fat percentage (n = 5/group; *p<0.05).
(TIF)

S5 Fig Effect of TGF-β inhibition on neurocognitive function. **(A)** Mice were treated with PBS, Pan02, or Pan02+11D1.16.8 and tested for rotarod running speed, or **(B)** object recognition (n = 5/group; *p<0.05).
(TIF)
