# Supplementary figures and images for: TGF-β Blockade Reduces Mortality and Metabolic Changes in a Validated Murine Model of Pancreatic Cancer Cachexia
Source: PLoS One. 2015 Jul 14;10(7):e0132786. doi: 10.1371/journal.pone.0132786 (PMC4501823; doi:10.1371/journal.pone.0132786)

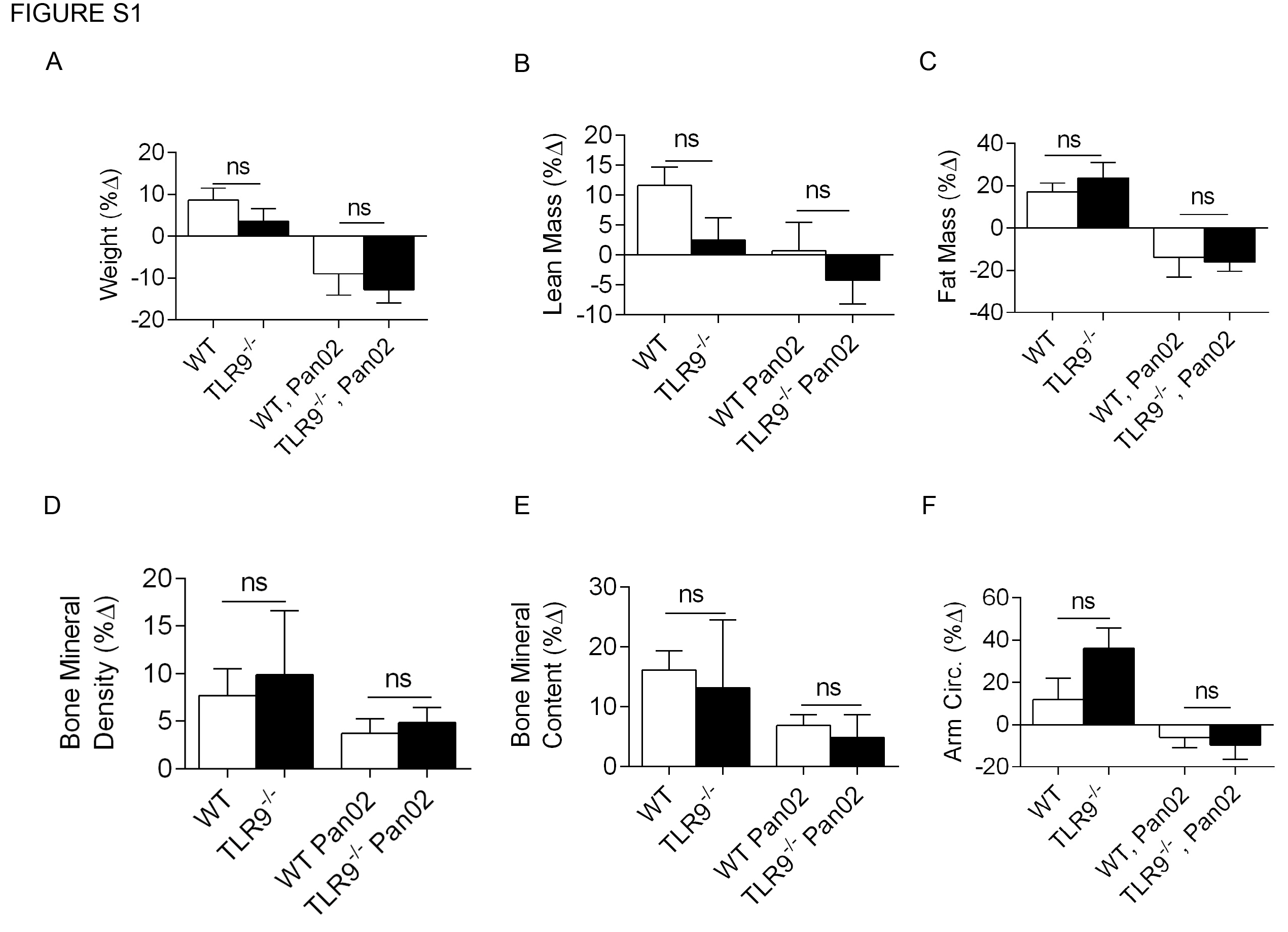

Supplement: S1 Fig — (A) WT and were treated TLR9-/- mice were treated with PBS or Pan02 and tested for overall weight change, and changes in (B) lean body mass, (C) fat mass, (D) bone mineral density, (E bone mineral content, and (F) arm circumference (n = 10/group). (TIF) [file pone.0132786.s001.tif]

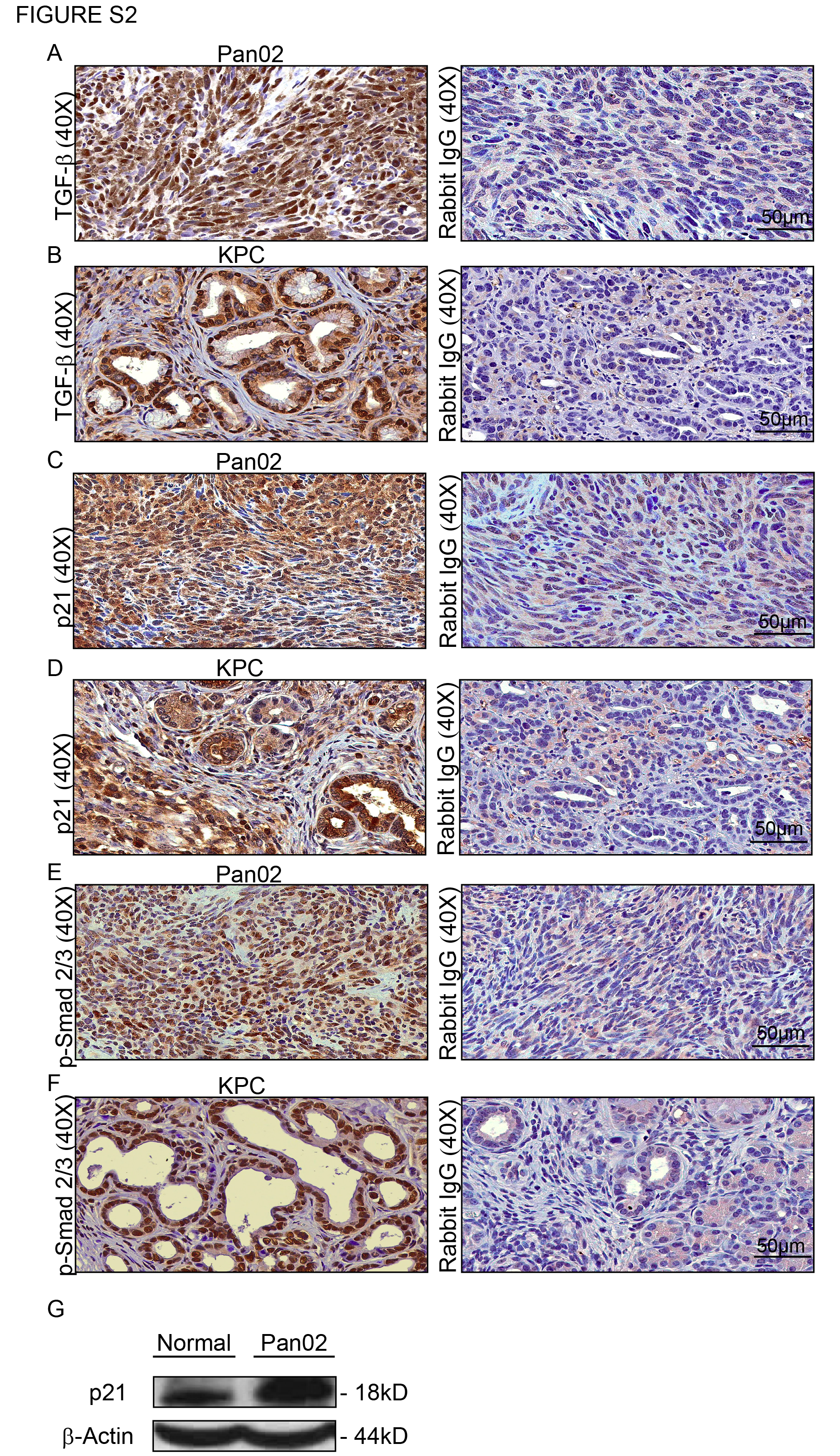

Supplement: S2 Fig — (A) Expression of TGF-β was tested by immunohistochemistry in Pan02 peritoneal carcinomatosis and (B) in the pancreata of KPC mice. (C) Expression of p21 was tested by immunohistochemistry in Pan02 peritoneal carcinomatosis and (D) in the pancreata of KPC mice. (E) Expression of p-Smad2/3 was tested by immunohistochemistry in Pan02 peritoneal carcinomatosis and (F) in the pancreata of KPC mice. (G) Protein levels of p21 in quadriceps muscle of mice treated with PBS and Pan02 were tested by Western blotting (n = 5/group). (TIF) [file pone.0132786.s002.tif]

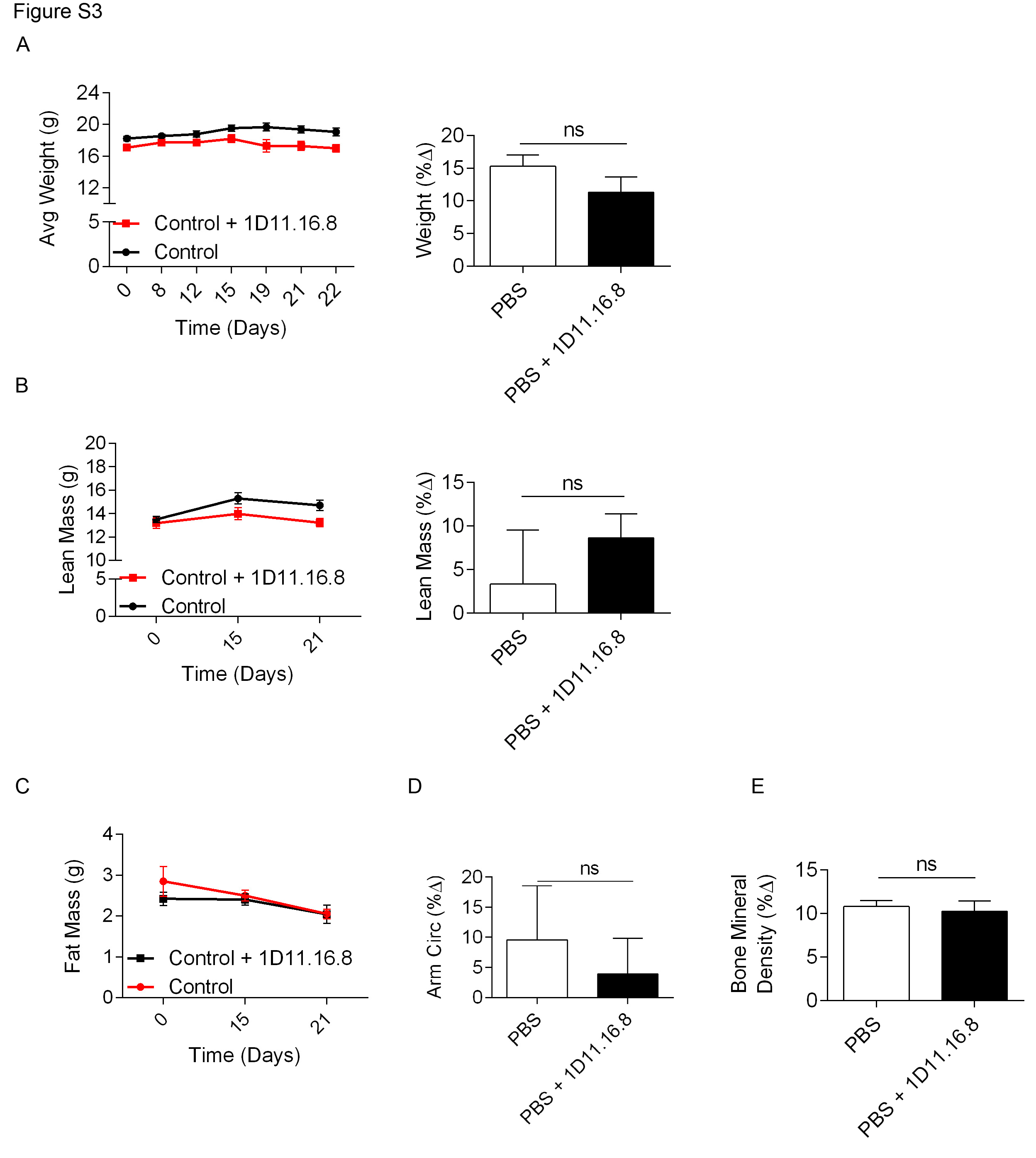

Supplement: S3 Fig — (A) Mice were treated with PBS or 11D1.16.8 and tested for weight change, and changes in (B) lean mass, (C) fat mass, (D) arm circumference, or (E) bone mineral density (n = 10/group). (TIF) [file pone.0132786.s003.tif]

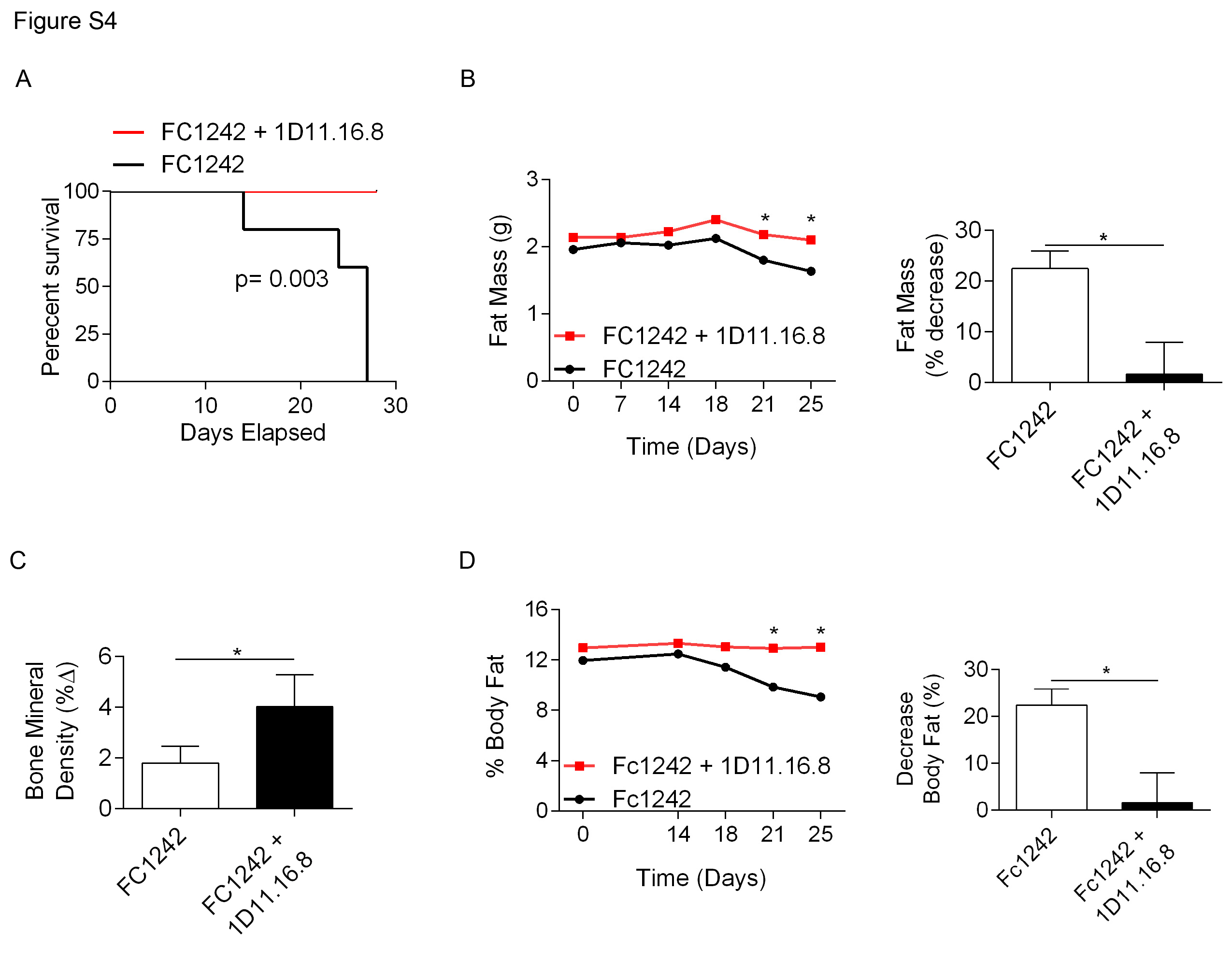

Supplement: S4 Fig — (A) Mice were treated with FC1242 or FC1242 + 11D1.16.8 and tested for survival using Kaplan-Meier analysis. (B) Cohorts of mice were also tested for change in fat mass, (C) change in bone mineral density, and (D) body fat percentage (n = 5/group; *p<0.05). (TIF) [file pone.0132786.s004.tif]

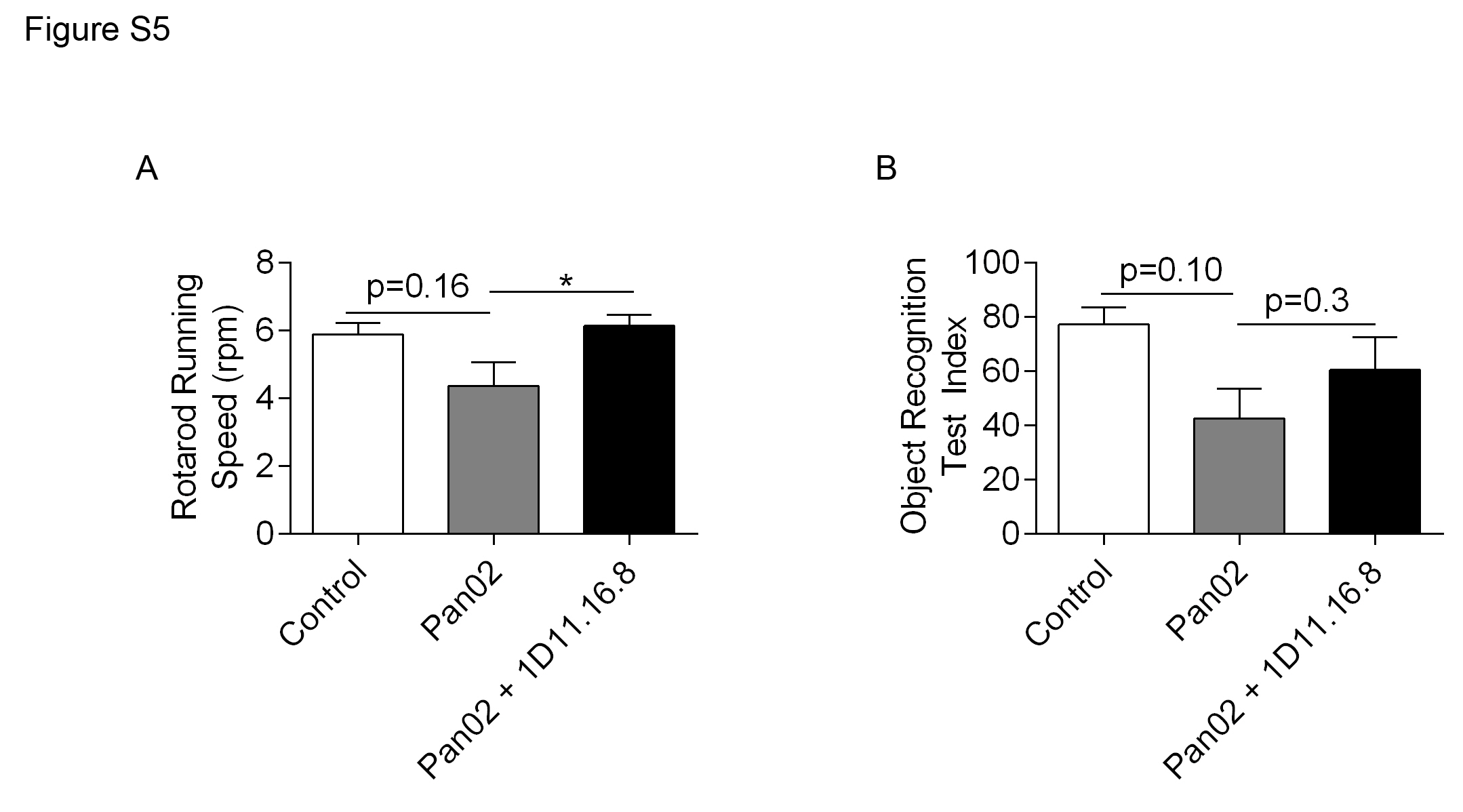

Supplement: S5 Fig — (A) Mice were treated with PBS, Pan02, or Pan02+11D1.16.8 and tested for rotarod running speed, or (B) object recognition (n = 5/group; *p<0.05). (TIF) [file pone.0132786.s005.tif]
